# Supramolecular Copolymerization of Bichromophoric Chiral and Achiral Perylenediimide Dyes

**DOI:** 10.3389/fchem.2021.652703

**Published:** 2021-03-26

**Authors:** Shumpei Yonezawa, Tsuyoshi Kawai, Takuya Nakashima

**Affiliations:** Division of Materials Science, Graduate School of Science and Technology, Nara Institute of Science and Technology, Ikoma, Japan

**Keywords:** chiral assembly, chiral amplification, circular dichroism, supramolecular copolymerization, prochiral

## Abstract

Propagation and amplification of chirality are considered to play an important role in the chemical evolution of biological homochirality. Stereochemical communications have been demonstrated to have a significant effect on the formation of chiral hierarchical structures in helical polymers, surface assemblies and supramolecular polymers. The formation of supramolecular copolymers based on chiral and achiral bichromophoric perylenediimide (PDI) dyes having a binaphtyl- and biphenyl-core-bridging unit, respectively, was investigated in terms of chiral amplification and propagation. The biphenyl-bridged PDI dye was expected to perform as a prochiral component to adopt both right- and left-handed twisting structures with the free rotation over the phenyl-phenyl linkage upon partnered with the chiral binaphtly PDI dye in the coassemblies. The coassemblies between the chiral and achiral PDI dyes with dissimilar core units demonstrated the composition dependent control in the length of supramolecular nanofibers as well as amplification of optical activity.

## Introduction

The origin of biological homochirality has been subject to debate and intrigued scientists for a long time. While it is not clear if the homochirality is a requisite property for biological activity, benefits of chirality have been recognized in some technological applications (Brandt et al., 2017). For example, homochirality was reported to confer an advantage to the mechanical property of oligopeptide-based hydrogels (Taraban et al., 2012). The hydrogels composed of a pair of oppositely charged peptides with the same chirality afforded the higher elastic modulus than the ones with heterochiral peptide pairs. This result evokes the role of homochirality in collagen fibers in which the chirality propagates over hierarchy to construct collagen superhelices, leading to the unique mechanical properties of collagen tissues (Kadler et al., 2007).

Even achiral small molecules with extended *π*-conjugated aromatic units such as perylene (Li et al., 2006), triphenylene (Kimura et al., 2010) and benzocoronene (Zhang et al., 2013) often self-assemble into twisted nanofibers. One-directional rotation bias in *π*-stacking to avoid the steric hindrance between side-chain groups (Babu et al., 2014) leads to the supramolecular chirality. In other words, the unidirectional chirality translation and the one-dimensional growth of supramolecular polymer take place in a synchronized manner. However, such the chiral supramolecular polymerization of achiral components mostly results in the formation of racemic mixtures aside from unexpected occurrence of symmetry breaking (Liu et al., 2015). Introduction of a small amount of a chiral component in an assembly of achiral or prochiral molecules with an analogous structure successfully guides the one-handed global supramolecular chirality resulting in the chirality amplification as a consequence of “sergeants-and-soldiers” principle (Palmans et al., 1997; Smulders et al., 2008). Chiral sergeant molecules and achiral soldier molecules share a common central self-assembling core unit with aromatic rings in the most cases of chirality amplification experiments, while the former component only tethers side-chains with point chirality. Such the chiral units in the periphery of the molecules successfully dictate the handedness in the rotational stacking of aromatic rings. However, the expression of sergeants-and-soldiers principle in the coassembly of chiral and achiral molecules with dissimilar self-assembling core units has been rarely demonstrated (Liu et al., 2015).

**FIGURE 1:**
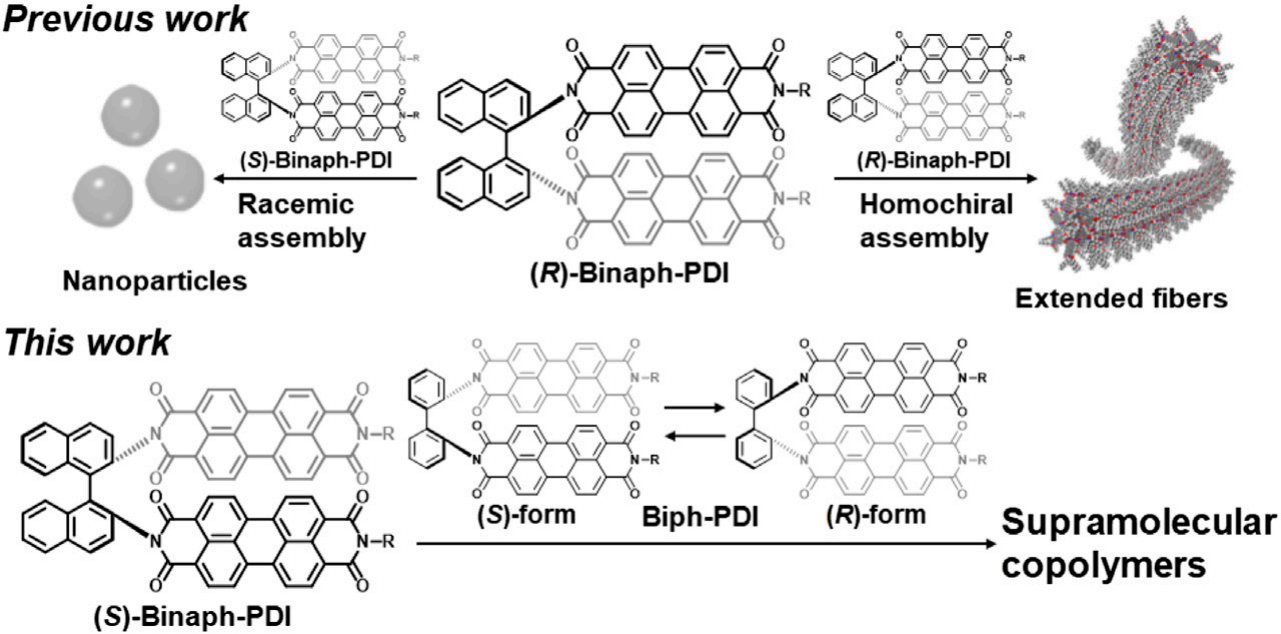
Shape-recognition mediated self-assembly of Binaph-PDI and its coassembly with Biph-PDI [R = CH–(C_6_H_13_)_2_].

While the chirality at periphery directs the rotational direction in monomer stacking with the aid of *π* interactions between aromatic cores, the chirality at core such as axial chirality has a direct impact on the supramolecular growth with one-handed twist (Henderson and Castellano, 2021). The chiral cores including helicene (Lovinger et al., 1998; Kaseyama et al., 2011) and binaphthalene (Kumar et al., 2015) exhibit a self-chiral-recognition capability in *π*-stacked assemblies owing to their twisted geometry. For example, a bichromophoric binaphtyl derivative bearing two PDI units (Binaph-PDI, Figure 1) demonstrated an enantiopurity-dependent supramolecular polymerization behavior (Kumar et al., 2015). The molecule was originally synthesized by Langhals (Langhals and Gold, 1997) and its circularly polarized luminescence (CPL) performance was explored by us (Kawai et al., 2007). The enantiopure compounds was then found to form extended nanofibers with one-handed helical twist in a methylcyclohexane (MCH)-based solvent (Kumar et al., 2014). In the enantiopurity-dependent supramolecular polymerization, the coassemblies of scalemic mixture led to the shortening of nanofibers with decreasing the enantiopurity, resulting in the formation of particle assembles for the racemic composition (Kumar et al., 2015). The heterochiral stacks between the binaphtyl-cores with opposite chiral twists in a face-to-face manner were considered to terminate the one-dimensional growth of chiral supramolecular polymerization. Although such the preference of heterochiral assembly to homochiral one was observed, Binaph-PDI exhibited the “majority-rules” effect (van Gestel et al., 2005) in the heterochiral assembly, wherein the major enantiomeric component dominates the global supramolecular chirality to some extent. In the present study, we investigate the coassembly of chiral Binaph-PDI with an achiral bichromophoric-PDI dye, Biph-PDI (Figure 1), possessing the dissimilar core. Given the prochiral structure of biphenyl core, Biph-PDI could serve as both (quasi-)enantiomeric forms when partnered by Binaph-PDI for the coassembly. Two stories are likely applied for the coassembly of Binaph-PDI and Biph-PDI. Biph-PDI could adopt a twisting structure opposite to that of Binaph-PDI to form quasi-heterochiral stacks, terminating the supramolecular polymerization of Binaph-PDI. Meanwhile, Biph-PDI could be incorporated in the chiral assembly of Binaph-PDI, participating in the chiral supramolecular copolymerization with an identical screw sense. We thus demonstrate a sort of modified “sergeants-and-soldiers” experiment through the coassembly of Binaph-PDI and Biph-PDI as a chiral and achiral (or prochiral) component, respectively.

## Results and Discussion

**FIGURE 2:**
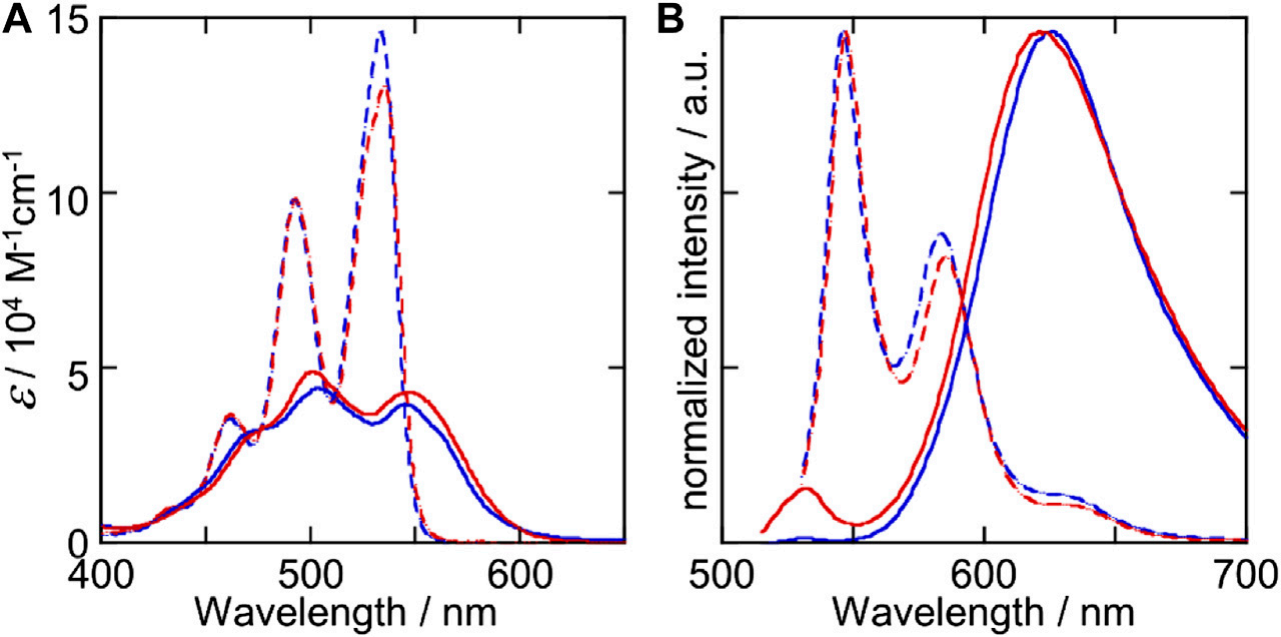
**(A)** Absorption and **(B)** fluorescence spectra of (*S*)-Binaph-PDI (red lines) and Biph-PDI (blue lines) in chloroform (broken lines) and in a mixture (1:19) of chloroform/MCH (solid lines).

Figure 2 compares the absorption and fluorescence spectra of (*S*)-Binaph-PDI and Biph-PDI in chloroform, in which both the compounds were molecularly dispersed. While both the molecules afforded very similar absorption and fluorescence spectra, Biph-PDI gave a more pronounced 0-0 transition band at 534 nm in absorption and a very slight blue-shift in emission, suggesting the less overlap of PDI units compared to Binaph-PDI (Kumar et al., 2013). Because of the less intramolecular interaction between the PDI units, Biph-PDI exhibited the slightly higher fluorescence quantum yield of 91% than that of Binaph-PDI (88%, Kawai et al., 2007). This difference should be attributed to the difference in the rotatability over the aryl-aryl bond in the core unit. Binaph-PDI with the more restricted rotatability between the naphthalene units led to the slightly larger interaction between the PDI units compared to Biph-PDI.

**FIGURE 3:**
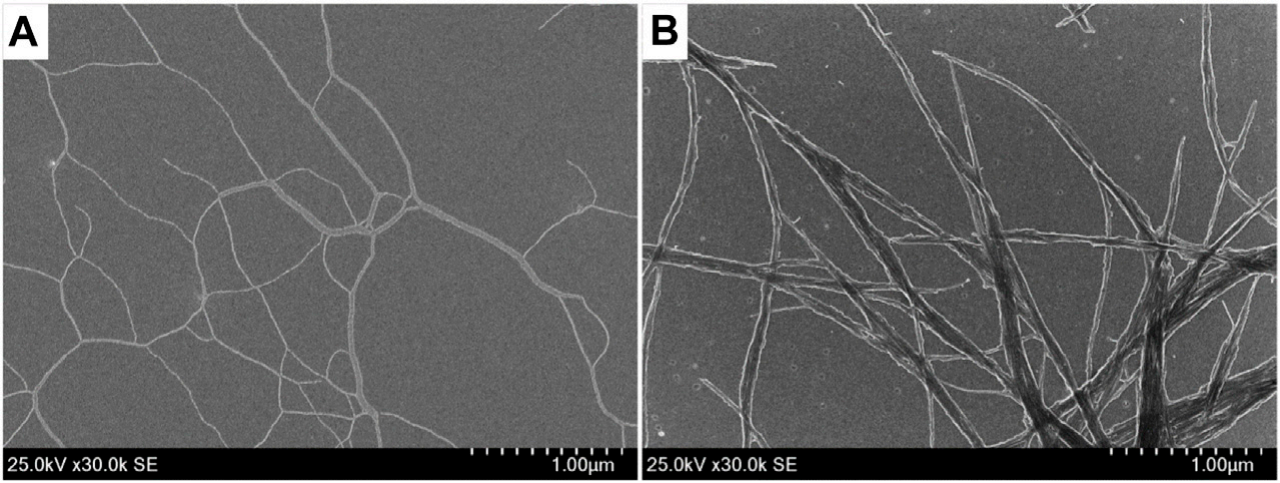
SEM images of self-assemblies of **(A)** (*S*)-Binaph-PDI and **(B)** Biph-PDI.

Several solvent systems were surveyed for the self-assembly of Binaph-PDI and a methylcyclohexane (MCH)-based solvent system was found to be most suitable (Kumar et al., 2014). Scanning electron microscopy (SEM) measurements disclosed that both the bichromophoric PDI-derivatives exhibited supramolecular assembly in a mixture (1:19) of chloroform/MCH with fibrous morphologies (Figure 3A) (*S*)-Binaph-PDI afforded extended and flexible nanofibers of a length over 5 μm with a width of 4.5 nm (Supplementary Figure S1) which is a bit wider than the unimolecular length, suggesting a cylindrical assembly with a rotational stacking of binaphtyl-PDI core as reported previously (Kumar et al., 2015). The nanofibers formed by achiral Biph-PDI appear to be less flexible with a thicker width of 7.5 nm (Supplementary Figure S1) and are more likely to form bundles in comparison to (*S*)-Binaph-PDI (Figure 3B). The difference in the rotatability of core biaryl units indicated by the absorption and emission spectra (Figure 2) should have an effect on the difference in the molecular ordering in their assemblies, leading to the apparent difference in the appearance of fibers and secondary aggregation property (bundling). The self-assembled morphology of Biph-PDI was also different from that of the racemic mixture of (*R*/*S*)-Binaph-PDI (Kumar et al., 2015).

Absorption spectra of the self-assemblies in chloroform/MCH solutions are also similar for (*S*)-Binaph-PDI and Biph-PDI (Figure 2A), that are typical for aggregates composed of PDI units with suppressed absorptivity (Reine et al., 2020). The absorption band at 550 nm typical for the homochiral assembly of Binaph-PDI (Kumar et al., 2014) slightly shifted to 547 nm for the self-assembly of Biph-PDI together with a slight appearance of a shoulder at 565 nm, which was absent for (*S*)-Binaph-PDI. Both the assemblies gave an emission band at around 620 nm typical for PDI assemblies whereas the Biph-PDI assembly exhibited the smaller emission quantum yield (Φ_f_) of 27% than that of (*S*)-Binaph-PDI (41%). The more pronounced *p*-π stacking with closer distance in the self-assembly of Biph-PDI, which is also suggested by the more suppressed molar absorptivity in Figure 2A, could contribute to the effective quenching of excited state by photophysical processes such as charge separation and biexcitonic annihilation, resulting in the less Φ_f_ value (Fuller et al., 2005). The supramolecular polymerization of each compound was considered to follow an isodesmic model, in which each step of the monomer attachment to the assembly end is governed by a single equilibrium constant, *K* (Chen et al., 2007). This result well accords with the self-assembly mechanism for the molecules composed of Ar-PDI core with alkyl-chains, wherein the *p*-π stacking between the Ar-PDI units and their solvophobic effect in the MCH-based solvent operate as the driving forces of self-assembly. The analysis of concentration dependent absorption spectra based on this isodesmic model led to a reasonable estimation of association constants *K* to be 1.2 × 10^5^ and 5.1 × 10^5^ M^−1^ for (*S*)-Binaph-PDI and Biph-PDI, respectively (Supplementary Figure S2).

**FIGURE 4:**
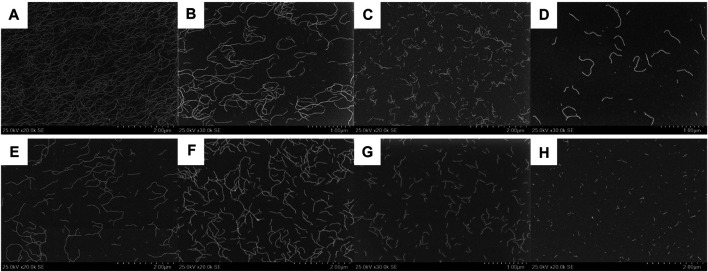
SEM images of (*S*)-Binaph-PDI-Biph-PDI coassemblies with the (*S*)-Biph-PDI content of **(A)** 90, **(B)** 80, **(C)** 70, **(D)** 60, **(E)** 50, **(F)** 40, **(G)** 30, and **(H)** 20 mol%.

We then examined the supramolecular copolymerization of Biph-PDI and (*S*)-Binaph-PDI with varied mixing ratios. The chloroform solutions of compounds were mixed with certain ratios and the mixed solution was diluted by MCH to give a chloroform/MCH (1:19) solution with the total concentration of 3.0 × 10^–5^ M. The mixture solutions were heated above 95 C for 5 min followed by slow cooling to room temperature. Very interestingly (*S*)-Binaph-PDI-Biph-PDI coassemblies formed the shorter fibrous morphologies while each the homo-component self-assembly afforded extended longer nanofibers (Figures 3, 4). The average length of nanofibers became shorter to > 1 μm, 790 and 330 nm with increasing the Biph-PDI content to 10, 20 and 30 mol%, respectively (Figure 4 and Supplementary Figure S3). The fiber length then increased to ca. 500 nmat 40 and 50 mol% Biph-PDI contents, which again decreased to 400, 270 and 130 nm with increasing the Biph-PDI content to 60, 70 and 80 mol%, respectively. The SEM images of coassembly with 90 mol% Biph-PDI content included both the short nanofibers of coassembly and extended fibers corresponding to the homoassembly of Biph-PDI (Supplementary Figure S4). Meanwhile, the thickness of nanofibers also differs dependent on the mixing ratio. The nanofiber width of coassemblies with the Biph-PDI contents of 10–30 mol% was similar to that of homo-component assembly of (*S*)-Binaph-PDI (4.5 nm), while those for coassemblies with the Biph-PDI content over 40 mol% were thicker (Supplementary Figure S5). All the coassemblies with the Biph-PDI contents of 40–80 mol% possessed a similar thickness of 6.5 nm (Figures 4D–H and Supplementary Figure S5). To summarize the morphological change in response to the coassembly composition, two types of structures are observed (nanofiber A, NF-A) shorter nanofibers with the width of 4.5 nm for coassemblies with the Biph-PDI contents of 10–30 mol% and (nanofiber B, NF-B) thicker nanofibers of 6.5 nm width for ones with the Biph-PDI content of 40–90 mol%.

The shortening of nanofibers was demonstrated for the heterochiral assembly of (*R*/*S*)-Binaph-PDI with decreasing the enantiopurity, in which the racemic assembly gave nanoparticles (Kumar et al., 2015). The decrease in the fiber length from the homochiral (*S*)-Binaph-PDI to NF-A with increasing the Biph-PDI content to 30 mol% may be attributed to the similar coassembling behavior to the heterochiral (*R*/*S*)-Binaph-PDI ones. The prochiral Biph-PDI behaves like the opposite enantiomer of (*S*)-Binaph-PDI with adopting left-handed twist. The stacks of molecules with opposite twists prevent the one-dimensional growth of supramolecular polymer with one-handed twisting orientation, resulting in the length shortening in NF-A. The NFs-B of ca. 500 nm length with the thicker width were then formed for the coassemblies with nearly even mixing ratios of (*S*)-Binaph-PDI and Biph-PDI (40–50 mol% Biph-PDI). With increasing the Biph-PDI content, the length of NF-B shortened. It should be noted that the increase in the purity of Biph-PDI which forms extended nanofibers led to the decrease in the length of nanofiber. These results observed for NF-B clearly suggest that each component adopts a packing structure different from each the homo-component assembly.

**FIGURE 5:**
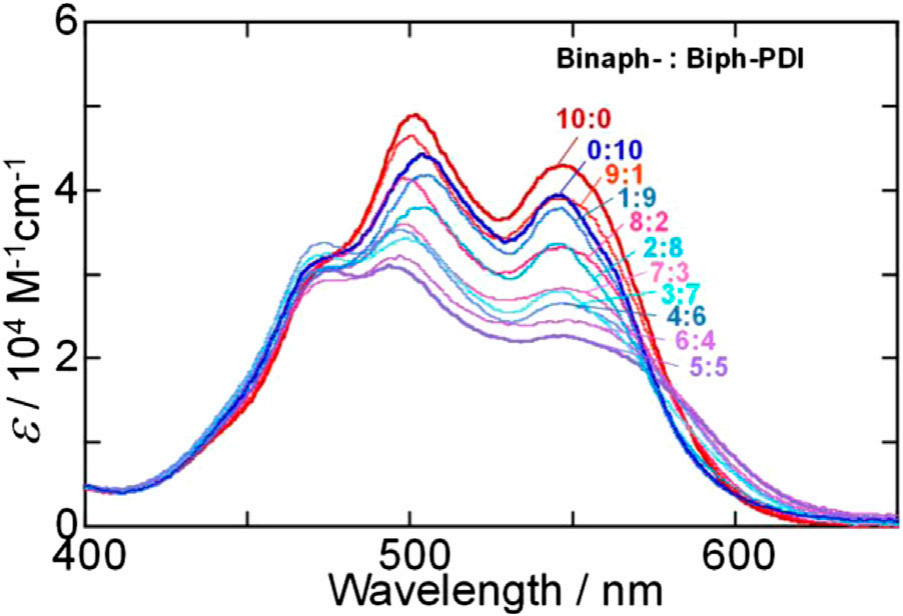
Absorption spectral change of Biph-PDI-(*S*)-Binaph-PDI coassemblies in a mixture (1:19) of chloroform/MCH [Biph-PDI] + [(*S*)-Binaph-PDI] = 3.0 × 10^–5^ M.

The absorption spectral change with changing the mixture ratio of (*S*)-Binaph-PDI and Biph-PDI also disagrees with the possibility of self-sorting but suggests the coassembly involving PDI-arrangements different from each homo-component self-assembly (Figure 5). That is, the absorption spectra of the mixtures could not be reproduced by a simple combination of absorption profiles of (*S*)-Binaph-PDI and Biph-PDI homo-component self-assemblies in line with the result of SEM observation. It is interesting to note that the molar absorptivity of assemblies decreases as the purity of each component decreases (Supplementary Figure S6). Especially, the 1:1 coassembly, corresponding to NF-B, afforded the broadest absorption profile with the most suppressed molar absorptivity. Thus the change in the absorption spectra suggests the change in the PDI-PDI interactions or the arrangement of PDI units in the coassemblies. The estimation of apparent association constant for the 1:1 coassembly led to the *K* value of 2.5 × 10^5^ M^−1^, which is inbetween those of homo-component assemblies (Supplementary Figure S2). The fluorescence quantum yield of coassemblies also afforded suppressed values with decreasing the purity of each component (Supplementary Figure S7). The coassemblies with the more pronounced hypochromic effect suggested the *π* stacking interactions with the closer distance, which should lead to the more suppressed fluorescence efficiency.

**FIGURE 6:**
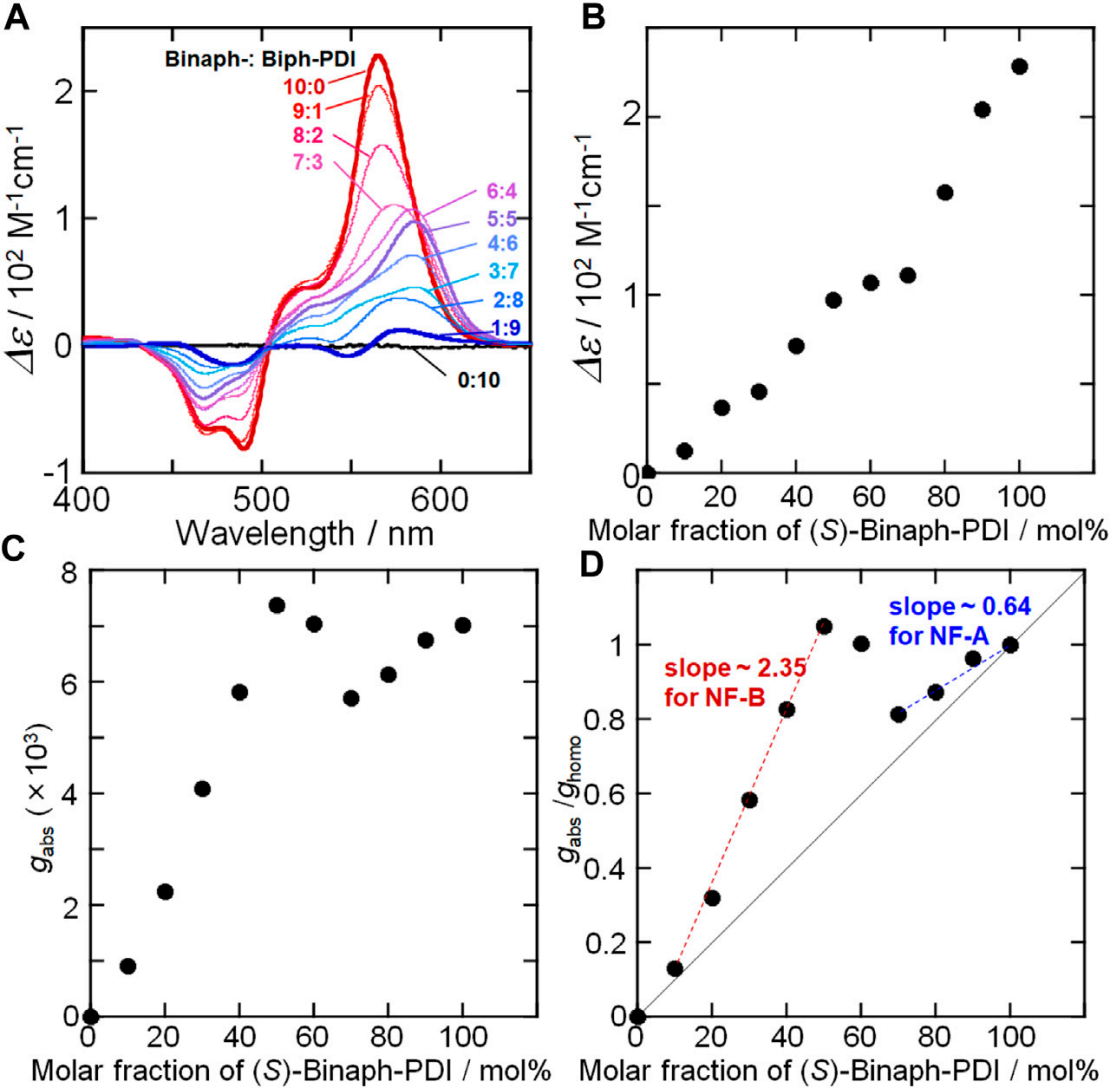
**(A)** CD spectral change of Biph-PDI-(*S*)-Binaph-PDI coassemblies in a mixture (1:19) of chloroform/MCH **(B)–(D)** Plots of molar CD, *g*_abs_ and *g*_abs_/*g*_homo_ values as a function of molar fraction of (*S*)-Binaph-PDI.

The CD spectral change was also investigated for the coassemblies of Binaph-PDI and Biph-PDI. The homo-assembly of (*S*)-Binaph-PDI afforded an apparent bisignate couplet with the first positive Cotton effect at 560 nm, suggesting the exciton coupling between PDI units in the assembly (Figure 6A). Upon mixing with achiral Biph-PDI, the CD amplitude (*Δε*) gradually decreased with increasing the molar fraction of Biph-PDI. The mirror image response was obtained for the (*R*)-Binpah-PDI-Biph-PDI coassemblies (Supplementary Figure S8). No apparent “sergeants-and-soldiers” effect could be recognized in the plot of molar CD (*Δε*) at the peak of the first Cotton effect as a function of molar fraction of chiral (*S*)-Binaph-PDI (Figure 6B). However, a chiroptical response unique to the chiral-achiral coassembly with the dissimilar self-assembling cores was found in the changes of CD profile (Figure 6A). The addition of 40 mol% Biph-PDI resulted in a noticeable red-shift of the positive CD peak from 560 to 585 nm, suggesting the change in the chiral excitonic interaction between the PDI units. The shift in the chiral PDI assembly structures in response to the chiral-achiral component ratio was also suggested by the plot of *g*_abs_ (*g*_abs_ = *Δε*/*ε*) value (Figure 6C). By the dilution of (*S*)-Binaph-PDI assembly with the addition of achiral Biph-PDI, the *g*_abs_ value decreased until the Biph-PDI content of 30 mol% but suddenly increased at 40 mol%. This (6:4)-(*S*)-Binaph-PDI-Biph-PDI ratio corresponds to the composition at which the nanofiber morphology changes from NF-A to NF-B. Then the *g*_abs_ values for the Binpah-PDI contents of 40, 50 and 60 mol% become higher than that of coassembly with higher enantiopurity (70 mol% (*S*)-Binaph-PDI content). These coassemblies with nearly even (*S*)-Binaph-PDI-Biph-PDI ratios (40–60 mol%-(*S*)-Binaph-PDI contents) exhibited the broad absorption spectra with the suppressed absorptivity together with the wider peak splitting of the bisignate couplet in CD profiles approximately corresponding to the Davydov splitting (Berova et al., 2007). Both these absorption and CD spectral properties supported the closer interchromophore (PDI-PDI) interactions for those coassemblies in NF-B. As the result, the 1:1 coasembly afforded the highest *g*_abs_ value regardless of 50% enantiopurity. To discuss the chirality amplification in the coassemblies, the observed *g*_abs_ value at the peak of first Cotton effect was normalized by the *g*_abs_ for the homochiral (*S*)-Binaph-PDI assembly (*g*_homo_) in Figure 6D. In this plot, all the values are above the theoretical line (solid line) assuming the linear contribution of (*S*)-Binaph-PDI proportional to its content, suggesting a certain degree of chiral amplification. Furthermore, the degree of amplification was roughly compared between in NF-A and in NF-B with the (*S*)-Binaph-PDI content over 70 and 10–50 mol%, respectively. The slope for the increase in *g*_abs_/*g*_homo_ value for NF-A and NF-B was compared as an amplification factor, which was estimated to be 0.64 and 2.35, respectively (Figure 6D). The more pronounced chirality amplification was suggested in NF-B than NF-A. This fact suggests that Biph-PDI unit served as a terminator of supramolecular polymerization in NF-A and mostly deteriorated the homochiral assembly of (*S*)-Binaph-PDI. On contrary, the PDI units attached to achiral Biph-PDI take part in the chiral PDI-arrangement together with the PDIs in (*S*)-Binaph-PDI to form NF-B with high chiroptical property.

**FIGURE 7:**
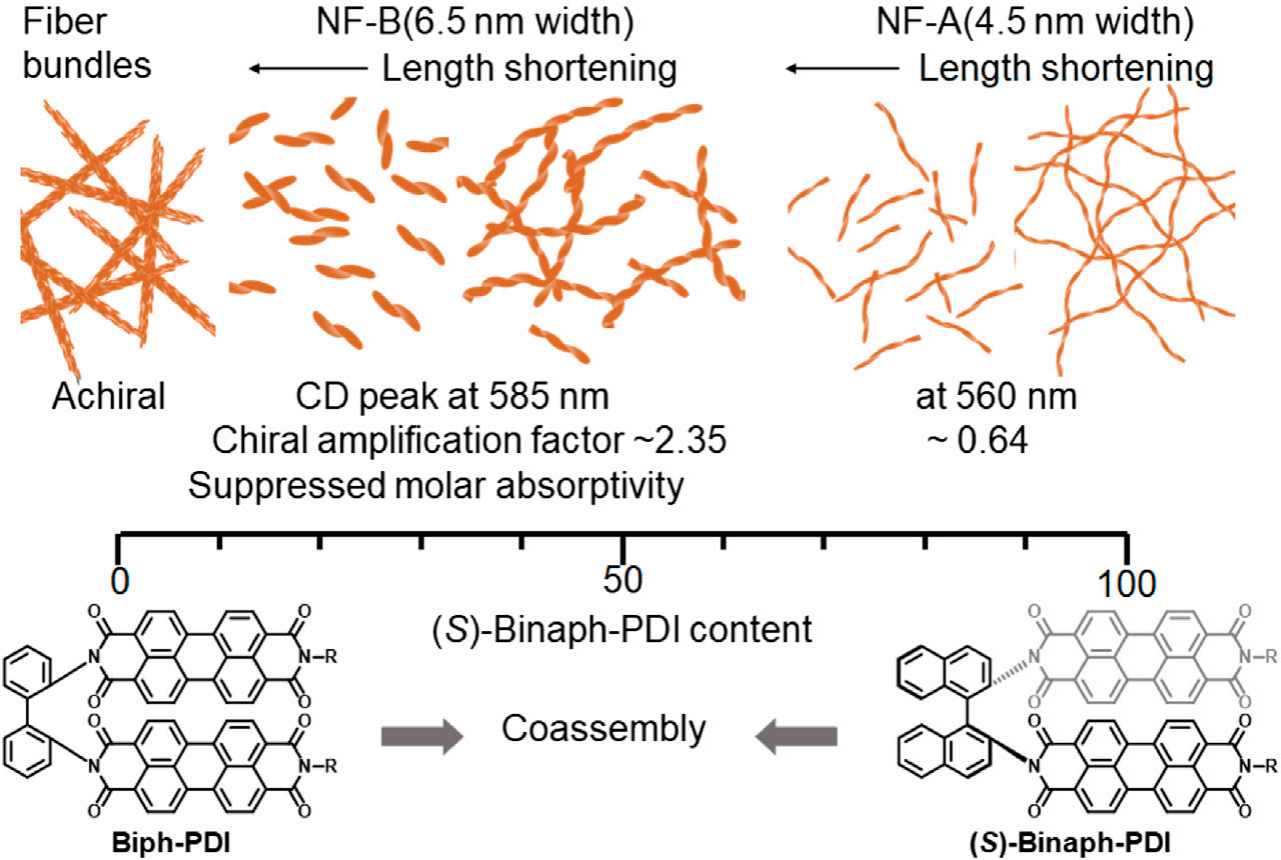
Schematic illustration of supramolecular copolymerization of Biph-PDI and (*S*)-Binaph-PDI.

We thus observed a nonlinear coassembling behavior in the supramolecular copolymerization between achiral Biph-PDI and chiral Binaph-PDI with dissimilar core structures. When chiral (*S*)-Binaph-PDI was the major component (>70 mol%), the self-assembling morphology was similar to that of homochiral assembly (NF-A). The powder X-ray diffraction study also supports this result (Supplementary Figure S9). The broad peak appeared around 20°–25° of 2*θ* could be assigned to the π stacking interactions between the Ar-PDI moieties in the assembly with the distance of 4.4–3.6 Å. The XRD profiles of the samples corresponding to NF-A gave the similar peaks to each other, suggesting the relatively loose *p*-π stacking interactions. The length of NF-A was shortened by increasing the content of achiral Biph-PDI, suggesting that the prochiral Biph-PDI molecule acts as a (*R*)-isomer to terminate the homochiral supramolecular growth of (*S*)-Binaph-PDI in a similar manner to the scalemic coassembly of (*R*/*S*)-Binaph-PDI (Kumar et al., 2015). Taking the fact that the *g*_abs_/*g*_homo_ values of NF-A were above the theoretical line into account, a part of Biph-PDI molecules were also incorporated in the NF-A with a one-handed screw sense. That is, even in the scalemic coasssembly of (*R*/*S*)-Binaph-PDI, the minor component molecules were incorporated into the fibers with a nonpreferred screw sense at the cost of mismatch penalty (Kumar et al., 2015). With further increasing the Biph-PDI content, Biph-PDI and (*S*)-Binaph-PDI coassembled to form NF-B with unique properties different from those in each homo-component assembly. The broad XRD peak shifted to the wider angle region suggesting the closer π stacking interactions. This result well accords with the wider peak splitting in the bisignate CD couplets for NF-B. Biph-PDI most likely participates in the chiral PDI arrangement in NF-B together with the chiral component of (*S*)-Binaph-PDI affording a high chiral amplification factor of 2.35. Even as the minor component (< 50 mol%) (*S*)-Binaph-PDI directs the global supramolecular chirality in NF-B involving the achiral Biph-PDI, thus serving as a sergeant in the supramolecular chirality amplification. In the absence of (*S*)-Binaph-PDI, Biph-PDI forms fiber bundles with no optical activity with a unique π stacking structure different from those of NF-A and NF-B (Figure 7, Supplementary Figure S9).

**SCHEME 1:**
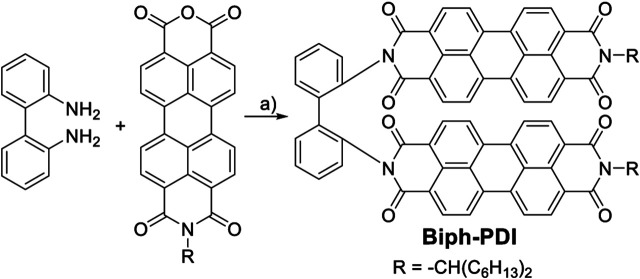
Synthetic route to Biph-PDI. Reagents and condition: A) 2,2′-biphenlydiamine (1 Eq.), N-(1-hexylhepthyl)-3,4:9,10-perylene tetracarboxylic anhydride imide (2 Eq.), imidazole as solvent, 170°C, 6 h (75%).

## Conclusion

In this study, we have investigated the supramolecular copolymerization behavior between an achiral and chiral PDI-dimer with different aryl-cores. Upon partnered with chiral Binaph-PDI, Biph-PDI was suggested to adopt different twisting geometries in the assemblies dependent on the coassembly composition. The adoption of different twisting structures in Biph-PDI led to the composition-dependent coassembly morphologies with different molecular stacks, demonstrating the protean nature of prochiral biphenyl bridging unit.

## Materials and Methods

### Synthesis of Compound Biph-PDI

*N*-(1-hexylhepthyl)-3,4:9,10-perylene tetracarboxylic anhydride imide (50.2 mg, 0.087 mmol) was reacted with 2,2′-biphenyldiamine (8.03 mg, 0.044 mmol) in imidazole (293 mg) at 170 C for 6 h under argon gas atmosphere. The reaction mixture was extracted with chloroform, washed with 1 M HCl aq. and brine and dried. The solvent was removed under reduced pressure and the residue was purified with column chromatography (CHCl_3_, silica gel) and a recycling preparative HPLC equipped with a gel-permeation chromatography column (CHCl_3_). The product was further purified using a HPLC with a normal phase silica gel column (CHCl_3_) to give the product as a red powder in 75% yield. The chemical structure was confirmed by mass spectrum and ^1^H and ^13^C NMR in CDCl_3_. ^1^H NMR (CDCl_3_, 500 MHz, TMS) δ 8.56–8.23 (m, 16H), 7.75 (d, 2H), 7.61 (t, 2H), 7.44 (t, 2H), 7.20 (d, 2H), 5.25–5.18 (m, 2H), 2.28 (s, 4H), 1.96 (s, 4H), 1.38–1.22 (m, 32H), 0.86 (s, 12H): ^13^C NMR (CDCl_3_, 126 MHz, TMS) δ 163.97, 162.69, 138.08, 134.76, 134.67, 134.27, 134.19, 133.76, 133.19, 131.54, 130.98, 129.64, 129.29, 129.02, 128.92, 128.51, 126.56, 126.15, 124.24, 123.34, 123.11, 122.87, 122.71, 54.91, 32.54, 31.86, 29.29, 27.12, 22.64, 14.04: MS (MALDI-TOF-MS) (m/z) [M + Na]^+^calcd. for C_86_H_78_N_4_O_8_Na^+^: 1317.571, found: 1317.571.

### Characterizations

NMR measurements were performed on a JEOL ECA-600. Mass spectra were measured using a JEOL JMS-S3000. The absorption spectra and fluorescence spectra were recorded on a JASCO V-760 spectrophotometer and a JASCO FP-8500 spectrofluorimeter, respectively. The absolute fluorescence quantum yields were determined by a Hamamatsu C9920–02. CD spectra were measured using a JASCO J-725 spectropolarimeter. SEM images were obtained using an ultra-high resolutions SEM SU9000 (Hitachi High-Tech, Corp) operated at 15–25 kV. Unstained specimens for SEM were prepared by dropping solutions of compounds onto carbon-coated copper grids. The XRD profiles were recorded using a Rigaku SmartLab X-ray diffractometer with Cu Kα radiation (*λ* = 0.154 nm).

## Data Availability

The original contributions presented in the study are included in the article/Supplementary Material, further inquiries can be directed to the corresponding authors.
