# Supplementary material for: Supramolecular Copolymerization of Bichromophoric Chiral and Achiral Perylenediimide Dyes
Source: Front Chem. 2021 Mar 26;9:652703. doi: 10.3389/fchem.2021.652703 (PMC8032881; doi:10.3389/fchem.2021.652703)
Supplement: Supplementary file 1 [file datasheet1.pdf]

Supplementary Material

Supramolecular Copolymerization of Bichromophoric Chiral and Achiral Perylenediimide Dyes

Shumpei Yonezawa[1], Tsuyoshi Kawai[1] and Takuya Nakashima[1]

[1]Division of Materials Science, Graduate School of Science and Technology, Nara Institute of Science and Technology, Ikoma, Nara, Japan

Supplementary Figures

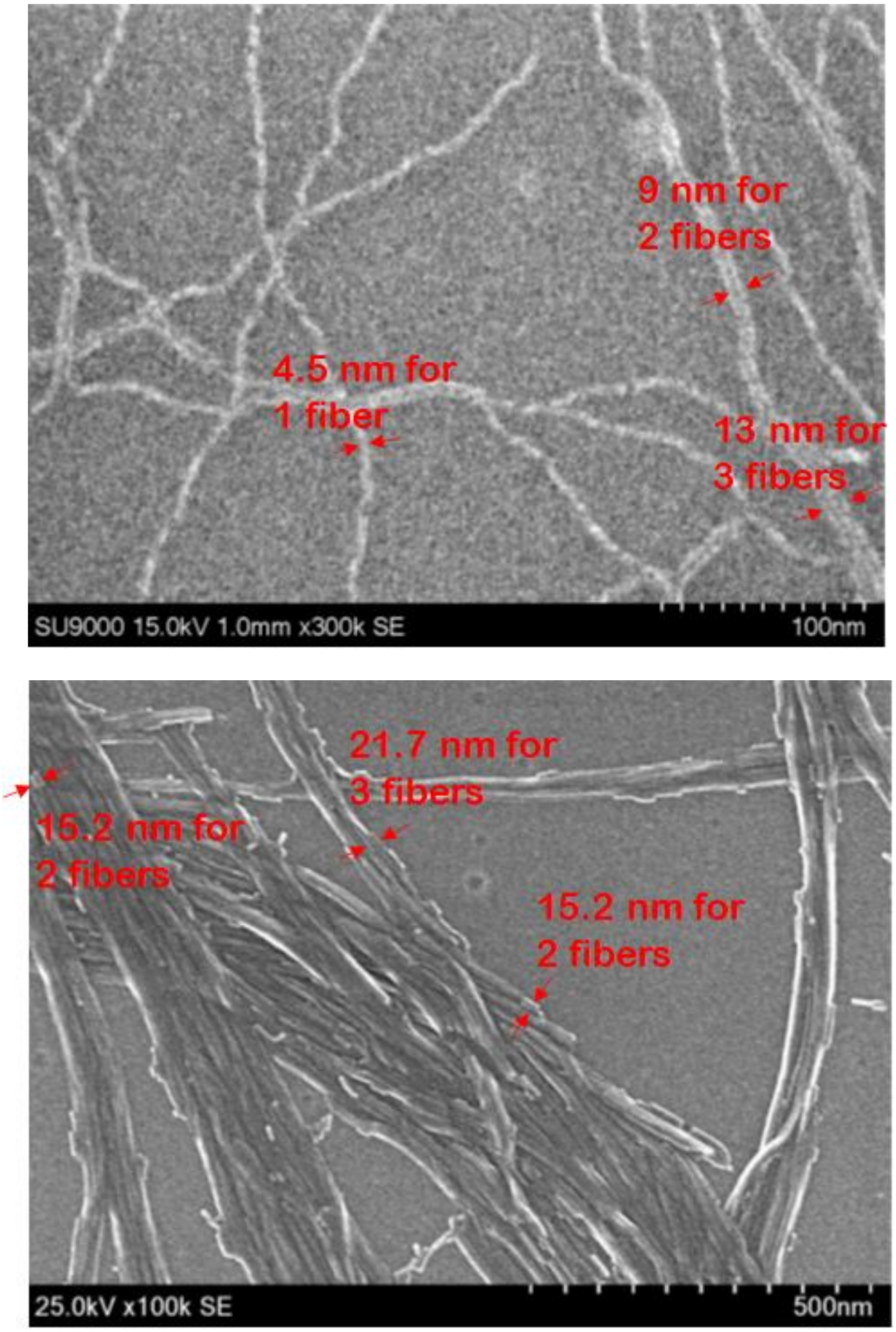

Figure S1. High magnification SEM images of self-assemblies of (top) (*S*)-Binaph-PDI and (bottom) Biph-PDI.

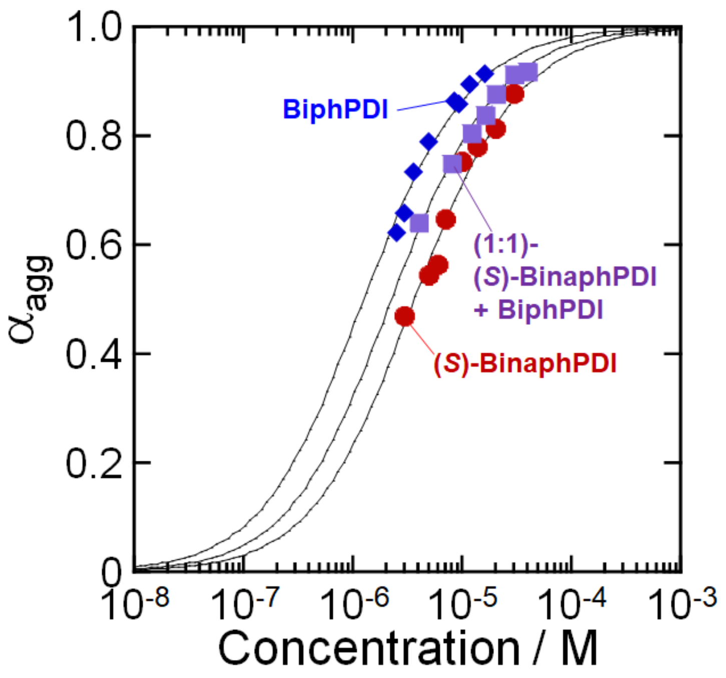

Figure S2. Plots of fraction of aggregated molecules (α_{agg}) as a function of total concentration of (*S*)-Binaph-PDI and Biph-PDI for the determination of association constant.

The fraction of aggregates (α_{agg}) and the association constants (*K*) was calculated by solving the following mathematical equations;

Mathematically the isodesmic model can be expressed by the equation [1]

$$\alpha_{agg} = 1 - \alpha_{mon} = 1 - c_{mon} / c_T = 1 - \frac{2Kc_T + 1 - (4Kc_T + 1)^{1/2}}{2(Kc_T)^2} \qquad (1)$$

where α_{mon} and α_{agg} are the fraction of molecules in the monomeric and aggregated states, respectively, c_{mon} is the concentrations of monomeric species, c_T is the total concentration in solution and *K* is the equilibrium constant.

This mathematical expression can be connected to absorption spectra resulting in eq 3

$$\alpha_{agg} = 1 - \frac{\varepsilon\,(c_T) - \varepsilon_{agg}}{\varepsilon_{mon} - \varepsilon_{agg}} \qquad (2)$$

$$\varepsilon\,(c_T) = \frac{2Kc_T + 1 - (4Kc_T + 1)^{1/2}}{2(Kc_T)^2}\,(\varepsilon_{mon} - \varepsilon_{agg}) + \varepsilon_{agg} \qquad (3)$$

where ε_{mon}, ε_{agg}, and $\varepsilon(c_T)$ are the molar absorption coefficients of free molecules in the monomeric state, aggregated state and the concentration cT, respectively.

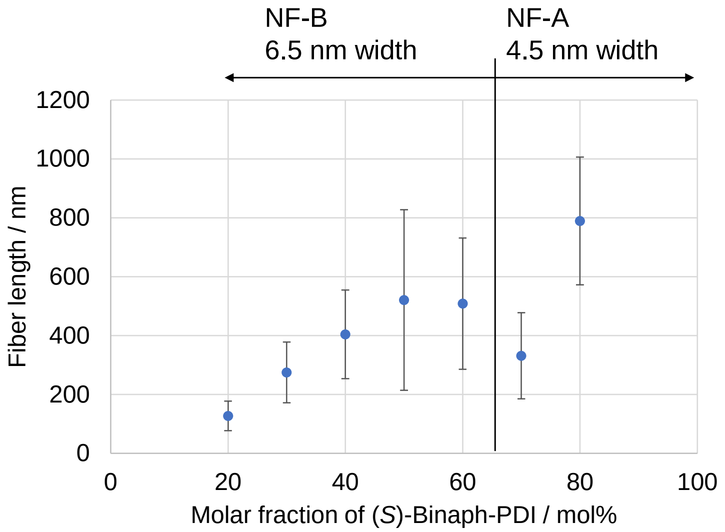

Figure S3. Plot of average fiber length of coassemblies as a function of (*S*)-Binaph-PDI contents.

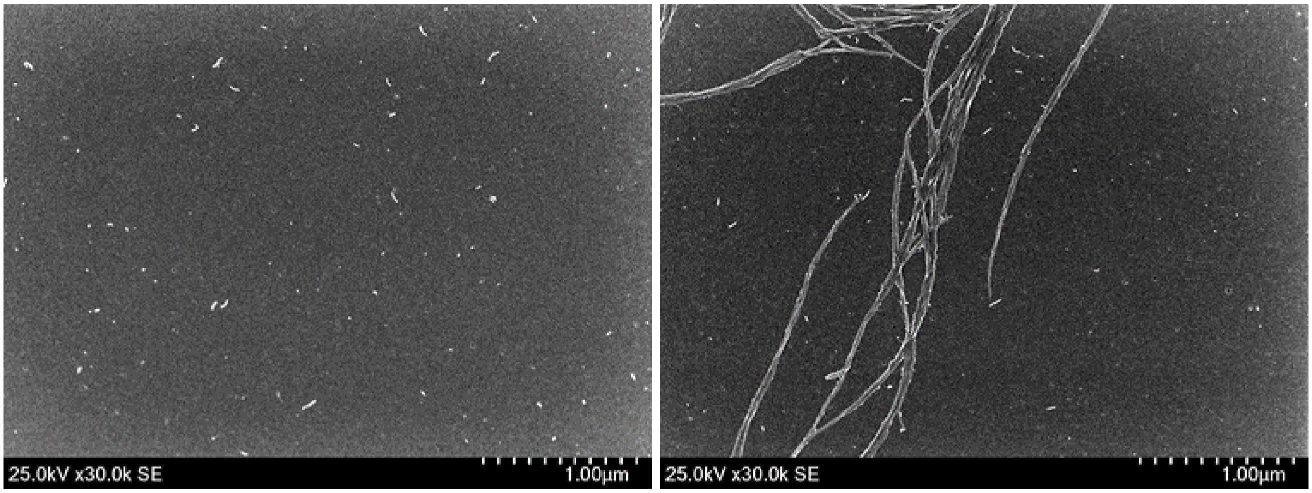

Figure S4. SEM images of (1:9)-(*S*)-Binaph-PDI-Biph-PDI coassembly.

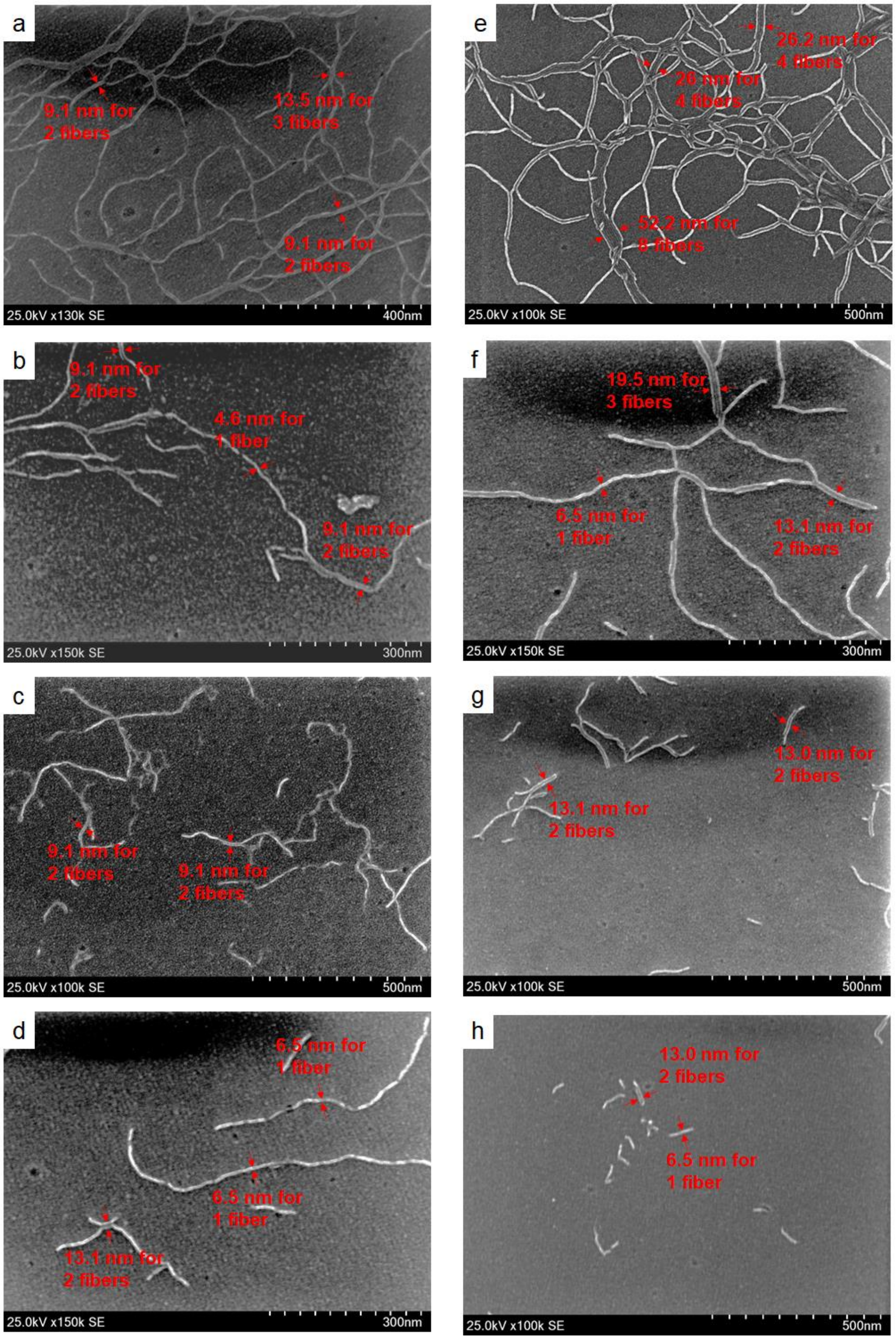

Figure S5. High magnification SEM images of (*S*)-Binaph-PDI-Biph-PDI coassemblies with Biph-PDI content of (a) 10, (b) 20, (c) 30, (d) 40, (e) 50, (f) 60, (g) 70 and (h) 80mol%.

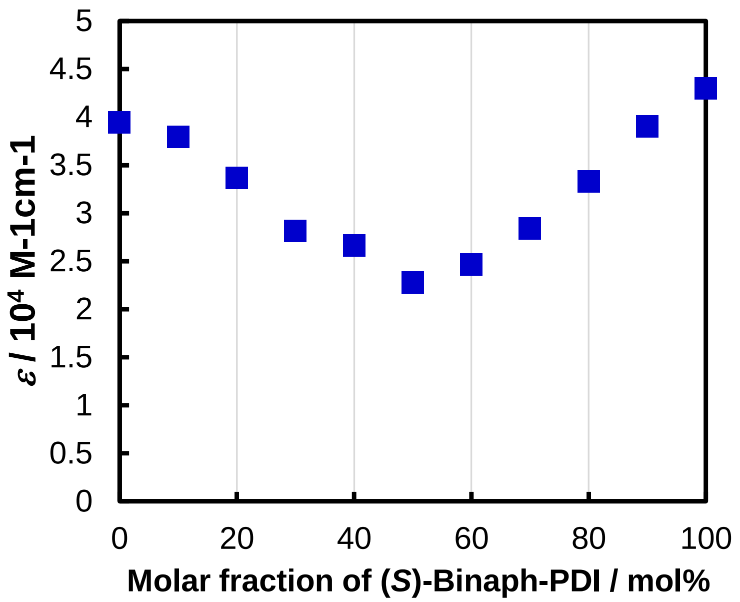

Figure S6. Plot of molar absorption coefficient at 550 nm of Biph-PDI-(S)-Binpah-PDI coassembly as a function of molar fraction of (S)-Binaph-PDI.

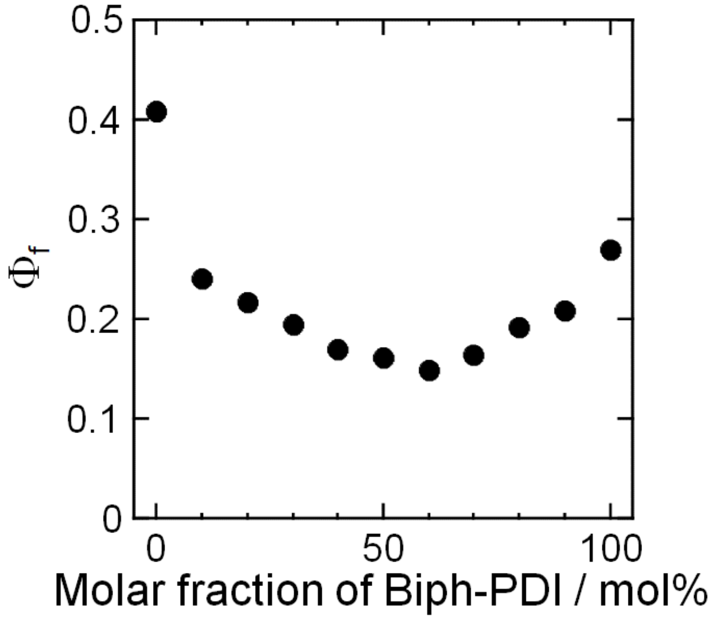

Figure S7. Plot of fluorescence quantum yield as a function of Biph-PDI content in the coassembly with (*S*)-Binaph-PDI in a mixture (1:19) of chloroform/MCH.

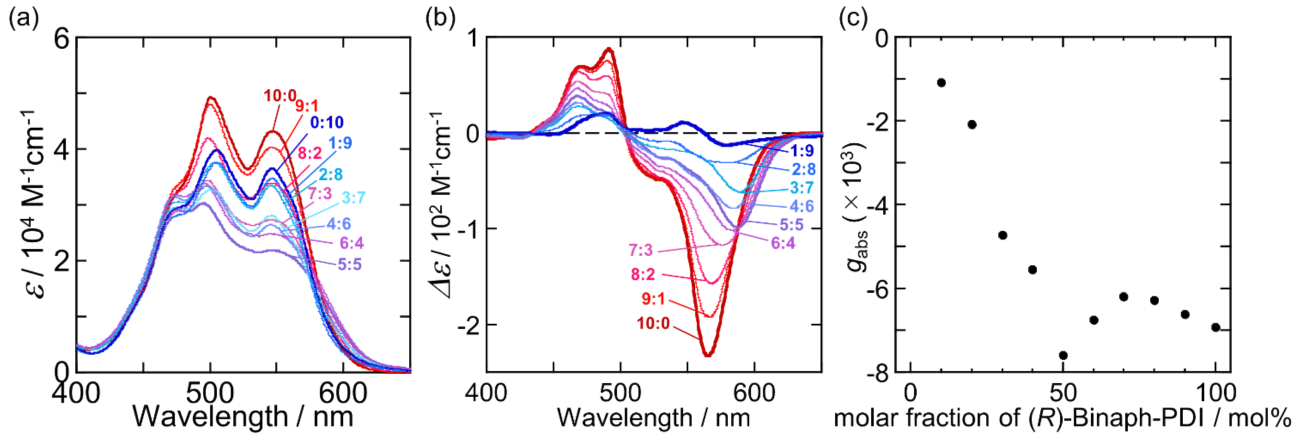

Figure S8. (a) Absorption and (b) CD spectral change of Biph-PDI-(*R*)-Binaph-PDI coassemblies in a mixture (1:19) of chloroform/MCH. [Biph-PDI]+[(*R*)-Binaph-PDI] = 3.0 × 10^{-5} M. (c) Plot of g_{abs} value as a function of molar fraction of (*R*)-Binaph-PDI.

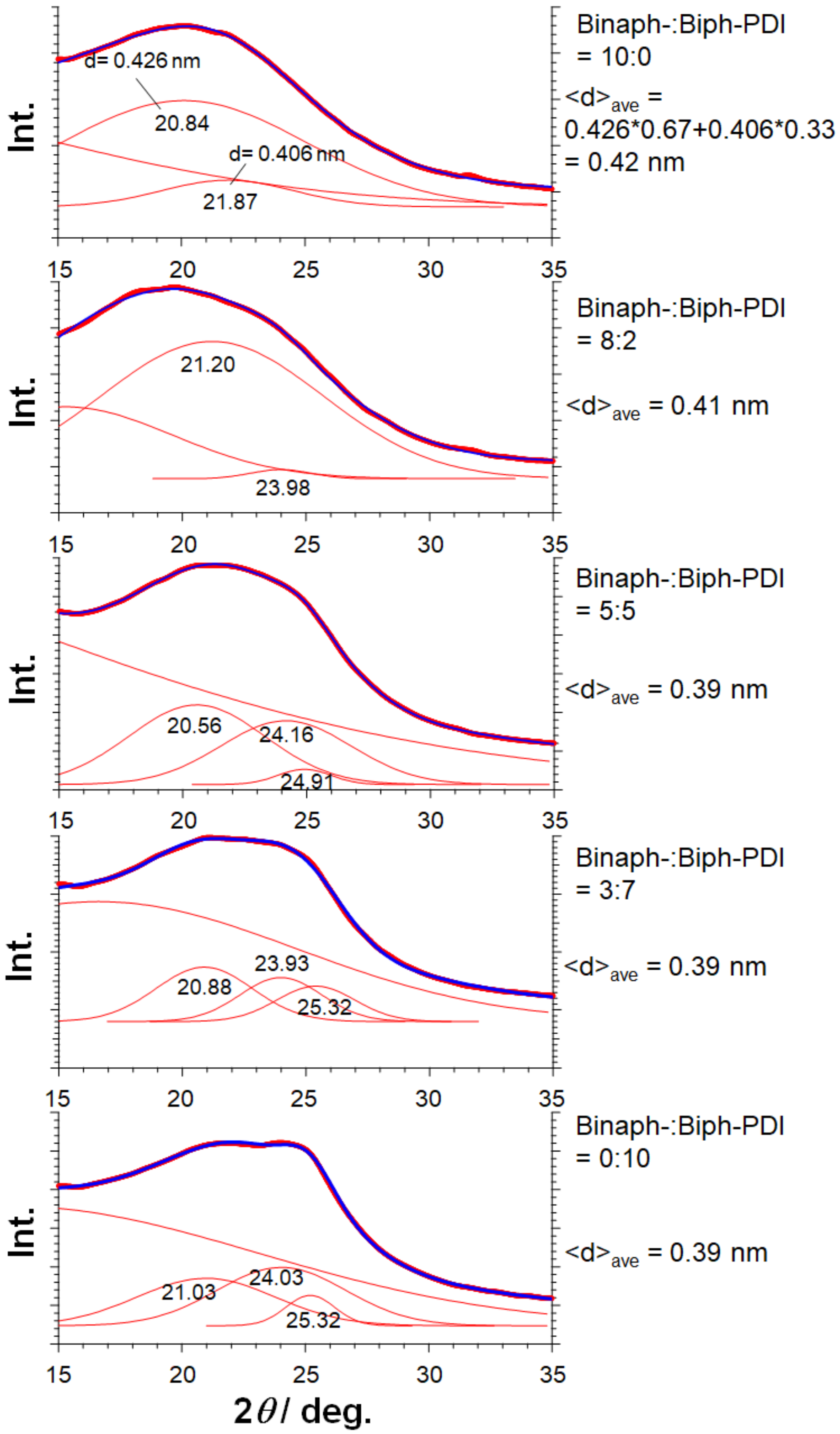

Figure S9. Powder XRD profiles of coassemblies of (*S*)-Binaph- and Biph-PDIs. Broad peaks were deconvoluted to 2 to 3 gaussian peaks. $<d>_{ave}$ was determined by considering the relative contribution of each peak.

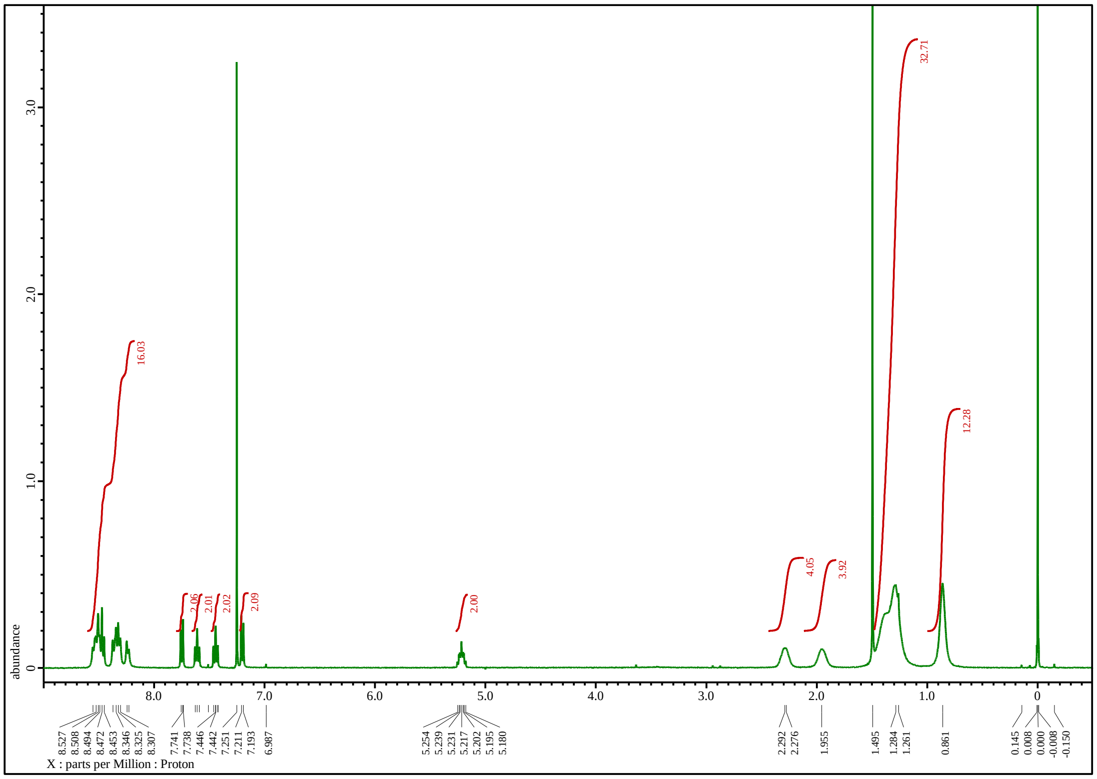

Figure S10. ^{1}H NMR spectrum of Biph-PDI ($CDCl_3$/TMS)

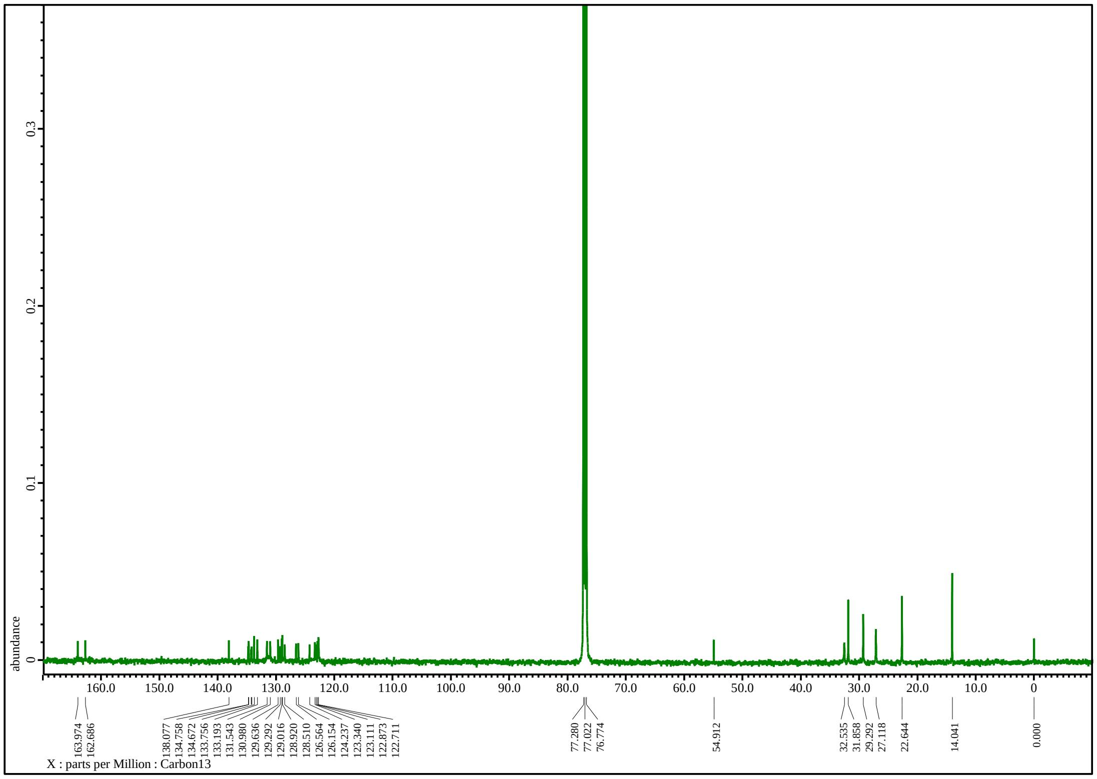

Figure S11. ^{13}C NMR spectrum of Biph-PDI ($CDCl_3$/TMS)

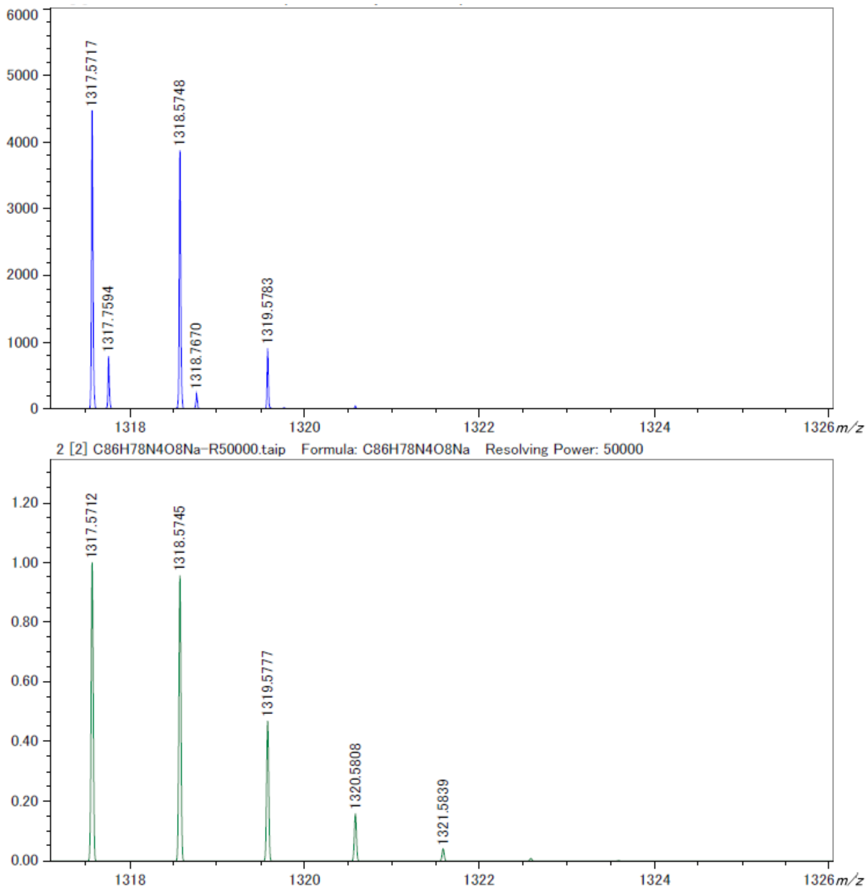

Figure S12. MALDI-MS spectrum of Biph-PDI (top) experimental result and (bottom) theoretical pattern.
